# The Mitochondrial Genome of a Plant Fungal Pathogen *Pseudocercospora fijiensis* (Mycosphaerellaceae), Comparative Analysis and Diversification Times of the Sigatoka Disease Complex Using Fossil Calibrated Phylogenies

**DOI:** 10.3390/life11030215

**Published:** 2021-03-09

**Authors:** Juliana E. Arcila-Galvis, Rafael E. Arango, Javier M. Torres-Bonilla, Tatiana Arias

**Affiliations:** 1 Corporación para Investigaciones Biológicas, Comparative Biology Laboratory, Cra 72A Medellín, Antioquia, Colombia; juearcilaga@unal.edu.co; 2 Escuela de Biociencias, Universidad Nacional de Colombia-Sede Medellín, Cl 59A Medellín, Antioquia, Colombia; rarango@cib.org.co (R.E.A.); javier.torres@colmayor.edu.co (J.M.T.-B.); 3 Corporación para Investigaciones Biológicas, Plant Biotechnology Unit, Cra 72A Medellín, Antioquia, Colombia; 4 Colegio Mayor de Antioquia, Grupo Biociencias, Cra 78 Medellín, Antioquia, Colombia

**Keywords:** banana, diversification times, mitochondrial genome, Mycosphaerellaceae, plant pathogens, *Pseudocercospora*, sigatoka disease

## Abstract

Mycosphaerellaceae is a highly diverse fungal family containing a variety of pathogens affecting many economically important crops. Mitochondria play a crucial role in fungal metabolism and in the study of fungal evolution. This study aims to: (i) describe the mitochondrial genome of *Pseudocercospora fijiensis*, and (ii) compare it with closely related species (*Sphaerulina musiva*, *S. populicola*, *P. musae* and *P. eumusae*) available online, paying particular attention to the Sigatoka disease’s complex causal agents. The mitochondrial genome of *P. fijiensis* is a circular molecule of 74,089 bp containing typical genes coding for the 14 proteins related to oxidative phosphorylation, 2 rRNA genes and a set of 38 tRNAs. *P. fijiensis* mitogenome has two truncated *cox1* copies, and bicistronic transcription of *nad2-nad3* and *atp6-atp8* confirmed experimentally. Comparative analysis revealed high variability in size and gene order among selected Mycosphaerellaceae mitogenomes likely to be due to rearrangements caused by mobile intron invasion. Using fossil calibrated Bayesian phylogenies, we found later diversification times for Mycosphaerellaceae (66.6 MYA) and the Sigatoka disease complex causal agents, compared to previous strict molecular clock studies. An early divergent *Pseudocercospora fijiensis* split from the sister species *P. musae* + *P. eumusae* 13.31 MYA while their sister group, the sister species *P. eumusae* and *P. musae,* split from their shared common ancestor in the late Miocene 8.22 MYA. This newly dated phylogeny suggests that species belonging to the Sigatoka disease complex originated after wild relatives of domesticated bananas (section Eumusae; 27.9 MYA). During this time frame, mitochondrial genomes expanded significantly, possibly due to invasions of introns into different electron transport chain genes.

## 1. Introduction

Mycosphaerellaceae is a highly diverse fungal family containing endophytes, saprobes, epiphytes, fungicolous and phytopathogenic species in more than 56 genera [1,2]. Family members can cause significant economic losses to a large number of important plants including ornamentals, food crops and commercially propagated trees [3,4,5,6,7,8]. Three Mycosphaerellaceae members, *Pseudocercospora eumusae*, *P. fijiensis*, and *P. musae*, [1] are major pathogens of bananas and plantains. They comprise the so-called Sigatoka disease complex which is responsible for one of the most economically destructive diseases for banana growers [9,10]. Diseases caused by these three pathogens induce plant physiological alterations including a reduction in photosynthetic capacity, crop yield, and fruit quality [9]. The Sigatoka disease complex causal agents form a robust clade, with *P. fijiensis* diverging earlier (39.9–30.6 MYA) than sister species *P. eumusae* and *P. musae* (22.6–17.4 MYA) [10,11,12]. Among them, *Pseudocercospora fijiensis* (teleomorph *Mycosphaerella fijiensis*) is the causal agent of black leaf streak disease (BLSD; aka Black Leaf Spot Disease), one the most damaging and costly diseases for banana and plantain worldwide [13].

Fungal mitochondrial genomes (mitogenomes) are circular or linear, usually AT enriched and range in size from 1.1 kb (*Spizellomyces punctatus*) [14] to 272 kb (*Morchella importuna*) [15]. Their size variation is mostly due to the presence or absence of accessory genes including RNA and DNA polymerases, reverse transcriptases and transposases, mobile introns, and size variation in intergenic regions [16,17]. In spite of the variation in size, their core gene content is largely conserved, even though their relative gene order is highly variable, both between and within the major fungal phyla [18,19,20]. Mitogenomes have introns and intronic open reading frames (ORFs) classified as group I and group II introns, which differ in their sequence, structure and splicing mechanisms [16,21,22,23,24,25]. Typically, group-II introns contain ORFs that code for reverse-transcriptase-like proteins. In contrast, group-I introns encode proteins with maturase and/or endonuclease activity [16]. Because of the limited comparative analysis of complete fungal mitogenome sequences, it has been difficult to estimate the timeframes and molecular evolution associated with mitochondrial genes or genomes [26].

Mitochondria have proven to be useful in evolutionary biology and systematics because they contain their own genome capable of independent replication, uniparental inheritance [27], near absence of genetic recombination, and uniform genetic backgrounds for some species [28]. Attempts to determine a time frame for fungal evolution are hampered by the lack of reliable fossil records. Hence, so far studies have focused on relating rates of DNA base substitutions and molecular clocks [29], based on the assumption that mutation rates of nuclear genes are similar to their counterparts in organisms with datable fossils [19]. Mitochondria plays a major role in fungal metabolism and fungicide resistance but until now only two annotated mitogenomes have been published in Mycosphaerellaceae (*Zasmidium cellare* and *Zymoseptoria tritici*) [30,31]. Sigatoka disease comparative mitogenome studies will provide answers on the evolution and adaptation of these plant pathogenic fungi.

This study aimed to: (i) sequence and characterize the complete mitogenome of *Pseudocercospora fijiensis*; (ii) compare mitogenomes of *P. fijiensis* with closely related species *P. eumusae,* and *P. musae* (causal agents of Sigatoka), and species with publicly available high throughput data such as *Sphaerulina musiva* and *S. populicola* (causal agents of leaf spot and canker diseases in poplar); and (iii) estimate timeframes and mitochondrial molecular evolution using fossil records to calibrate the Sigatoka disease complex phylogeny. We found that in mitogenomes analyzed herein, there were differences in content of free-standing and intronic Homing Endonuclease Genes (HEGs), genes coding for hypothetical proteins, and accessory genes such as DNA/RNA polymerases, reverse transcriptases and transposases. This work contributes to the understanding of mitogenome organization in Mycospharellaceae. In addition, new fossil calibrations for the Sigatoka’s complex species and mitochondrial comparative analysis aid in our understanding of the tempo and mode of evolution of these plant fungal pathogens.

## 2. Materials and Methods

### 2.1. Fungal Strain, DNA Extraction, and Library Construction and Sequencing

*P. fijiensis* isolate 081022 was obtained from naturally infected banana leaves coming from a commercial plantation located in Carepa, Antioquia, Colombia. Taxonomic affiliation has been confirmed based on both morphological criteria and Polymerase Chain Reaction (PCR) [32]. For DNA extraction mycelia from 7-day old culture were transferred to potato dextrose broth and incubated for 5–7 days at room temperature in a rotary shaker. Then, mycelia were harvested from the liquid using vacuum filtration. Total DNA was extracted using a previously described Cetyl Trimethylammonium Bromide (CTAB) method [33]. DNA quality and quantity were measured using a fluorometer (Qubit 3.0, Thermo Fisher Scientific, Waltham, MA, USA). Furthermore, genomic DNA was visualized on 1% agarose gel to check for any break/smear or multiple bands. Library construction was performed using Illumina platform with TruSeq DNA kit (Illumina, San Diego, CA, USA) to acquire as paired-end 2 × 150-bps, with about a 350-bp insert size. Next-generation sequencing was performed by an external service (North Carolina University, Chapel Hill, NC, USA) Hiseq 2500 system^®^.

### 2.2. Sequence Sources, Data Filtering and Assemblies

Eleven mitochondrial genome (mitogenomes) sequences were used for this study. Seven belonging to Mycosphaerellaceae: *Pseudocercospora fijiensis* (syn. *Mycosphaerella fijiensis*), *Pseudocercospora eumusae* (syn. *Mycosphaerella eumusae*), *Pseudocercospora musae* (syn. *Mycosphaerella musicola*), *Sphaerulina musiva* (syn. *Septoria musiva*), *Sphaerulina populicola* (syn. *Septoria populicola*), *Zasmidium cellare*, and *Zymoseptoria tritici* (syn. *Mycosphaerella graminicola*). One species is from Capnodiales: *Pseudovirgaria hyperparasitica* and three are from Pleosporales, the sister group of Capnodiales: *Didymella pinodes* (syn. *Mycosphaerella pinodes*), *Parastagonospora nodorum* (syn. *Phaeosphaeria nodorum*), and *Shiraia bambusicola* (Table S1). Mitogenomes were obtained either from our own sequencing data, or sequence data available at GenBank [34], RefSeq [35] or MycoCosm [36]. Authors, seq ID and databases are listed in Table S1.

Read quality was assessed using FastQC v. 0.11.5 [37] for the *P. fijiensis* isolate 081022 raw reads recovered here. Low-quality reads and/or bases were trimmed using Trimmomatic version 0.36 [38]. First, we de novo assembled whole DNA using Spades 3.9.0 (parameter “-careful”) [39] at different k-mer sizes (k = 61, 71, 81, and 91). The assembly with the highest N50 and assembly size was scaffolded by SSPACE version 3.0 [40]. Remaining gaps between scaffolds were closed using GapFiller version 1.10 [41] and a final genome assembly was evaluated by REAPR version 1.0.18 [42]. Scaffolds from the whole genome sequencing assembly were mapped to a draft and an unpublished *P. fijiensis* mitogenome available in MycoCosm [36] using Geneious 9.1.5 [43]. Mitogenomes were also filtered from de novo whole-genome assemblies for *Pseudocercospora musae* and *P. eumusae* available online [8,10]. To separate mitochondrial contigs or scaffolds from the nuclear contigs or scaffolds, we used BLASTn [44] and the Electron Transport Chain Conserved Mitochondrial Protein Coding Genes (CMPCGs) compiled from published mitogenomes of *Zasmidium cellare*, *Zymoseptoria tritici*, *Didymela pinodes*, *Phaeosphaeria nodorum* and *Sharaia bambusicola* as queries [30,31,45,46].

Even though the *Sphaerulina musiva* mitogenome was available online [8] we reassembled it using raw reads available at NCBI (SRA: SRR3927043). Our major motivation was a 9322 bp inversion detected around the 10,000 bp position of this mitogenome. This inversion was splitting the gene *nad2* and we wanted to make sure this inversion was present in the *S. musiva* mitogenome. First, raw reads were filtered using BBtools (https://sourceforge.net/projects/bbmap/ (accessed on 10 February 2021)) in Geneious 9.1.5 [43]. Then, MITObim version 1.8 [47] used *cox1* as bait to map all filtered reads that partly or fully overlap with the bait. Eventually, this leads to an extension of the reference sequence and a reduction of gaps until completion of the whole mitogenome [47]. This inversion in the *Sphaerulina musiva* mitogenome was found to be an artifact after reassembling raw reads.

Annotated mitochondrial genomes filtered from whole-genome assembly projects for *Pseudocercospora musae* and *P. eumusae* or reassembled for *Sphaerulina musiva* are available in Figshare (dataset: https://doi.org/10.6084/m9.figshare.12101058.v1 (accessed on 10 February 2021)).

### 2.3. Annotation

Mitochondrial genomes of *Pseudocercospora fijiensis*, *P. eumusae*, *P. musae*, *Sphaerulina populicola*, *S. musiva* and *Pseudovirgaria hyperparasitica* were annotated in this study using a combination of software. First, predicted ORFs were determined with a translation code for “mold mitochondrial genomes” using Geneious 9.1.5 [43]. Second, genes were identified using BLASTP version 2.4.0 [48] against the non-redundant protein database from NCBI (downloaded August and December 2016); genes were also identified using MITOS [49]. Third, protein domains and sequence patterns were searched with PFAM [50] and PANTHER 11.0 [51]. Additionally, mitogenome annotation was performed using multiple alignments among the fourteen CMPCGs using MUSCLE version 3.8.31 [52] and CLUSTAL W version 2.0 [53]. Inconsistencies regarding length and position of genes was solved paying particular attention to start and stop codons. Identified ORFs larger than 300 bp with start and stop codons that did not show results with the above-mentioned annotation strategies were considered as hypothetical proteins. Circular mitogenome maps were constructed using Geneious 9.1.5. and Geneious prime [43].

### 2.4. PCR Amplification of cox1 Gene Copies in P. fijiensis

A PCR assay was performed to confirm the presence of two different *cox1* copies: a truncated copy (*cox1_1*) and a complete *cox1* copy with an intron (*cox1_2*). Primers were designed to amplify regions between the first copy (*cox1_1*) and the second (*cox1_2*), including the exons of this last copy. First, a set of primers encompassed *cox1_2 exon1* and *cox1_2 exon 2*. A second pair of primers encompassed *cox1_1* and *cox1_2 exon 2*. PCR amplifications were carried out in a total volume of 10 μL, containing 20 ng genomic DNA, 0.15 μM of each primer, 1× PCR buffer (without MgCl_2_), 0.75 mM MgCl_2_, 4 μM of each dNTP and 0.65 U recombinant Taq DNA polymerase (Thermo Fisher Scientific, Waltham, Massachusetts, USA). Cycling parameters were: 3 min at 94 °C, followed by 35 cycles of 30 s at 94 °C, 30 s at a 50 to 60 °C temperature gradient to determine annealing temperature, 1 min at 72 °C, and a final elongation step of 5 min at 72 °C. PCR products were separated by electrophoresis in a 1% (*w*/*v*) agarose gel and visualized with GelRed^®^ (Biotium, Fremont, CA, USA) under UV light.

### 2.5. Transcriptome de novo Assembly

RNA-seq raw reads of *S. musiva* (SRR1652271) and *P. fijiensis* (SRR3593877, SRR3593879) were downloaded from the European Bioinformatics Institute (EMBL EBI) database. Reads were quality filtered and trimmed using BBDuck from BBtools (https://sourceforge.net/projects/bbmap/ (accessed on 10 February 2021)) before carrying out transcriptome de novo assemblies with Trinity version 2.3.1 [54]. The *P. eumusae* (GDIK00000000.1) and *P. musae* assembled transcriptomes (GDIN00000000.1) were also downloaded from GeneBank. RNA-seq Geneious 9.1.5. plugins were used to map the assembled transcripts to mitogenomes of either *S. musiva*, *P. fijiensis*, *P. eumusae* or *P. musae* paying particular attention to gene pairs *atp6-atp8*, *nad2-nad3*.

### 2.6. RT–PCR Assays for Mitochondrial Gene Pairs of P. fijiensis

Total RNA was extracted from *P. fijiensis* (isolate: 081022) mycelium after fifteen days of culture using TRIzol^®^ (Life Technologies, Carlsbad, CA, USA) according to the manufacturer’s instructions. RNA concentrations were measured at 260 nm using a NanoDrop ND-1000 UV-Vis Spectrophotometer (NanoDrop Technologies, Thermo Fisher). DNAse I (Thermo Fisher Scientific, Waltham, MA, USA) was used for cDNA synthesis from RNA as template for amplification using the Maxima First Strand cDNA Synthesis Kit (Thermo Fisher Scientific, Waltham, MA, USA) according to manufacturer’s instructions. Primers were designed such that the amplified product encompassed the end of one gene and the beginning of another. We used pairs of NADH-Ubiquinone Oxidoreductase Chain 3 and 2 (*nad3-nad2*) and mitochondrial encoded ATP Synthase Membrane Subunits 6 and 8 (*atp8-atp6*). Both genes and their intergenic sequences were partially amplified. PCR products were run in a 1% agarose gel electrophoresis purified using the GFX PCR DNA and gel band purification kit, according to manufacturer’s instructions (GE Healthcare, Chicago, IL, USA). Purified PCR products were sequenced using Sanger Technology at Macrogen Inc. (Seoul, Korea). All sequences are available in GeneBank: *atp6-atp8* cDNA (GenBank: MN171334); *atp6-atp8* DNA (GenBank: MN171335); *nad2-nad3* DNA (GenBank: MN171336); *nad2-nad3* cDNA (GenBank: MN171337); *cob* DNA (GenBank: MN171338); *cob* cDNA (GenBank: MN171339); *nad5* cDNA (GenBank: MN171340).

### 2.7. Identification of Repetitive Elements

Repetitive sequences in mitogenomes of Mycosphaerellaceae were identified and annotated using Geneious Primer Tandem Repeats Finder and using a minimum repeat length of 100, excluding repeats up to 10 bp longer [43]. Simple sequence repeat (SSR) markers and loci were identified using the MicroSAtellite Identification tool (MISA) [55] (https://doi.org/10.6084/m9.figshare.12101013 (accessed on 10 February 2021)).

### 2.8. Phylogenetic Analysis and Divergence Times Estimates

Until now, only nuclear markers and strict clock calibration have been used to calculate diversification times for the Sigatoka disease complex species. We aimed to compare these analyses with fossil calibrated Bayesian phylogenies and mitochondrial markers. A phylogenetic tree was reconstructed to calculate diversification times. Since most mitogenomes are uniparentally inherited we used our mitochondrial phylogeny to compare topologies with already published nuclear ones for the Sigatoka disease complex species. *Didymella pinodes* [56], *Pseudovirgaria hyperparasítica* [57], *Phaeosphaeria nodorum* [45] and *Shiraia bambusicola* [46] were used as outgroups. Ten core mitochondrial genes (*cox1*, *cox3*, *atp6*, *cob*, *nad1*, *nad2*, *nad4*, *nad4L*, *nad5*, *nad6*) were aligned one by one for all species using CLUSTAL W version 2.0 [53]. Then, all aligned genes were concatenated in a single alignment for phylogenetic reconstruction. *Cox2*, *atp8*, *atp9* and *nad3* were excluded from the alignment either because they could not be fully recovered in *P. musae* (*atp9*, *nad3*) or because they were missing in outgroups *S. bambusicola* (*atp8 atp9), P. nodorum* (*atp8*, *atp9*) and *D. pinodes* (*atp8*, *atp9*, *cox2*).

A Generalized time-reversible (GTR) model was used with an estimated gamma parameter of rate heterogeneity to build maximum likelihood (ML) trees using the Randomized Accelerated Maximum Likelihood RAxML version 8.0 [58] and PhyML version 3.0 [59] programs. One hundred bootstrapped trees were generated and used to assign bootstrap support values to the consensus trees. A Bayesian phylogeny and divergence time analysis was carried out using BEAST2 version 2.5.1 [60]. Separate partitions for each gene were created with BEAUti2 (available in BEAST2). More suitable substitution models for each gene were found using the software package jModelTest2 version 2 [61] according to the Bayesian Information Criterion (BIC) [62]. To accommodate for rate heterogeneity across the branches of the tree we used an uncorrelated relaxed clock model [63] with a lognormal distribution of rates for each gene estimated during the analyses. We also used a strict clock for further comparison of results.

The fossil Metacapnodiaceae [64] was used, assuming this to be a common ancestor of the order Capnodiales with a minimum age of 100 MYA (gamma distribution, offset 100, mean 180, maximum softbound 400). Capnodiales nodes were constrained to monophyly based on the results obtained from ML analysis. A birth/death tree prior was used to model the speciation of nodes in the topology, with gamma priors on the probability of splits and extinctions. We used vague priors on the substitution rates for each gene (gamma distribution with mean 0.2 in units of substitutions per site per time unit). All XML files used to build our Bayesian phylogenies are available at Figshare (https://doi.org/10.6084/m9.figshare.12101055.v1 (accessed on 10 February 2021)). To ensure convergence we ran analyses five times for 50 million generations each, sampling parameters every 5000 generations, assessing convergence and sufficient chain mixing (Effective Sample Sizes > 200) using Tracer version 1.5 [65]. After removal of 20% of each run as burn-in, the remaining trees were combined using LogCombiner (available in BEAST2), summarized as maximum clade credibility (MCC) trees in TreeAnnotator (available in BEAST2), and visualized using FigTree version 1.3.1 [66].

## 3. Results

### 3.1. P. fijiensis Mitochondrial Genome 

The whole-genome assembly of *P. fijiensis* had an N50 = 39,827 bp and a size of 70.53 Mb in 8040 scaffolds. Recovered scaffolds had an estimated genome coverage of 50X. To separate scaffolds belonging to *P. fijiensis* mitogenome, whole-genome scaffolds were mapped to a draft and unpublished *P. fijiensis* mitogenome (see Table S1). Scaffolds belonging to the mitogenome of *P. fijiensis* were recovered and assembled in a circular sequence deposited in GenBank (accession number: MK754071).

**Figure 1:**
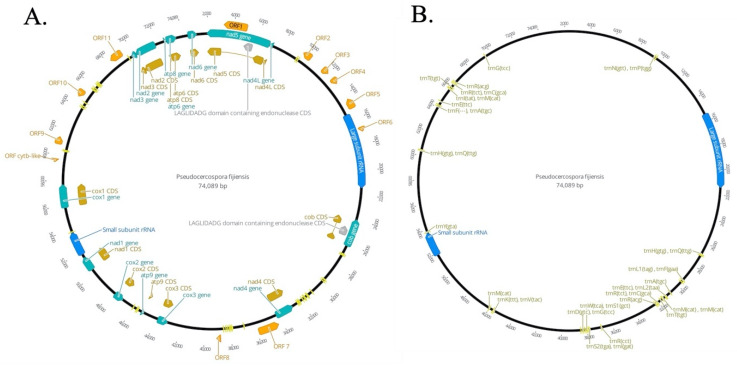
Circular map of the mitogenome of *Pseudocercospora fijiensis*. Genes are visualized by arrows, which are presented in a clockwise direction (forward). (**A**) Annotated introns, hypothetical Open Reading Frames (ORFs), group I mobile introns (Homing Endonuclease Genes (HEGs)) and genic regions; clusters of tRNA and rRNA are also indicated here. (**B**) Annotated tRNA and rRNA regions. Green color arrows: protein-coding genes; yellow color arrows: Coding regions CDS and introns; orange arrows: hypothetical ORFs; blue arrows: genes of large and small ribosomal subunits; bright yellow arrows: tRNAs genes; grey arrow: HEGs. Circular mitogenomes were generated by using the Geneious Primer [43].

*P. fijiensis* mitogenome is a circular molecule of 74,089 bp in length containing 14 Electron Transport Chain Conserved Mitochondrial Protein Coding Genes (CMPCG), two ribosomal RNAs, 38 tRNA genes and twelve putative Open Reading Frames (ORFs) of unknown function (Figure 1, Table 1). CMPCGs included ATP Synthase subunits (*atp6*, *atp8*, and *atp9*), Cytochrome Oxidase subunits I, II, and III (*cox1*, *cox2*, and *cox3*), Nicotinamide Adenine Dinucleotide Ubiquinone Oxidoreductase subunits *nad2* and *nad1*, *3*, *5*, *6*, *nad4L* (Figure 1A, Table 1).

Twelve putative ORFs of unknown function were predicted to produce hypothetical proteins containing 77 to 583 amino acids, which were described as ORF1-ORF11 and ORF *Cytb*-like. CMPCGs covered 65.55% of the genome (including 11.77% putative ORFs) (Figure 1A, Table 1). tRNA genes and both rnl and rns corresponded to 8.8% and 10.67% respectively (Figure 1B, Table 1). These values were similar to those reported for other representatives of the Ascomycota phylum (Table 2). Overall, *P. fijiensis* mtDNA has a nucleotide composition of: 36.6% of A, 13.4% of C, 12.6% of G and 37.7% of T. GC-content was 26% with coding and non-coding parts of the genome having, on average, the same GC-percentage.

The three most frequent codons were TTA (708 counts), ATA (498 counts) and TTT (498 counts) encoded amino acids leucine, isoleucine, and phenylalanine, respectively. These amino acids have hydrophobic side chains commonly found in transmembrane helices. These three codons accounted for 6.9% of all codons in the mitogenome. Eight codons (ATA, ATT, TTA, and TTG encoding methionine; CGA, CGC, CGG encoding arginine and TAG encoding a stop codon) were under-represented, being used from one to five times each. CMPCGs started with ATA, ATG, ATT or TTA encoding methionine translation initiation codon. The preferred stop codon was TAA, present in 21 protein-coding genes; the alternative stop codon was TAG. Codon usage of the ORFs was similar to that of the protein-coding loci. *Atp9* also contained the highest GC contents (40.9%) among all the CMPCGs (Table 1).

Thirty-eight tRNAs encoded by the mitogenome of *P. fijiensis* carry all 20 amino acids (Figure 1B, Table 1). Two tRNA iso-acceptors were identified for serine and leucine, and four for arginine and methionine. Among the 38 tRNAs, tRNA-Val, tRNA-Tyr, tRNA-Asp, tRNA-Lys, tRNA-Asn, tRNA-Pro occurred singly (Table 1). tRNA genes were grouped into nine clusters. (Figure 1B). 

Twenty-nine repeat regions ranging from 611 bp to 128 bp were located in non-coding regions of the mtDNA, but only one was present three times (length 193 bp) (Table 2). A search within the *P. fijiensis* mitogenome detected 33 SSR markers. The most common SSR were mononucleotide (30 SSR), and only three dinucleotide SSR were found (https://doi.org/10.6084/m9.figshare.12101058 (accessed on 10 February 2021)). A difference of seven nucleotides between *P. fijiensis* isolate 081022 and the unpublished mitochondrial genome available in MycoCosm was found, being 99% identical (Table S1). 

#### 3.1.1. Presence of Truncated Conserved Mitochondrial Protein-Coding Genes (CMPCGs)

**Figure 2:**
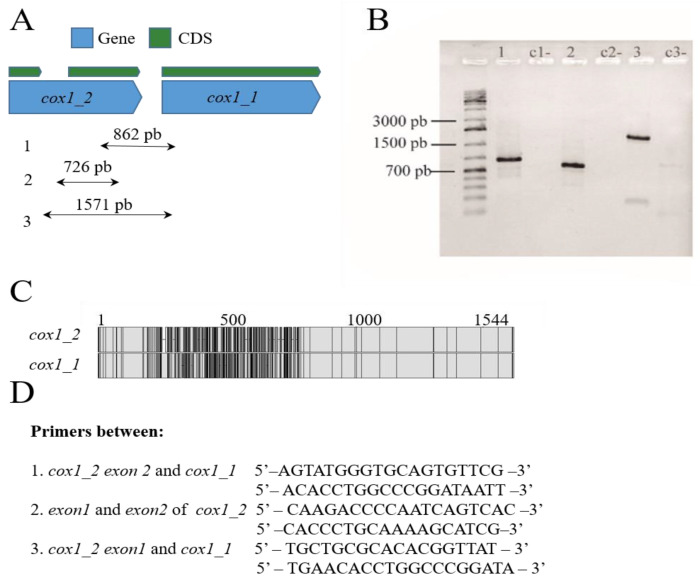
Duplicated Protein-coding genes in the mitogenome of *Pseudocercospora fijiensis*. (**A**) A truncated *cox1* copy and a complete copy with an intron that are localized in tandem. (**B**) PCR amplification including intra and intergenic regions between truncated *cox1* copies. (**C**) Pairwise alignments of truncated *cox1* copies reveal a region of around 600 bp of low similarity between them (non-identical sites appear in black in the alignment). (**D**) Primers used for the amplification of both *cox1* copies.

*P. fijiensis* mitogenome had truncated copies of four CMPCGs. They were considered truncated copies because they have PFAM and PANTHER domains and high local sequence similarity (>90%) with a complete CMPCG copy despite being an incomplete sequence without start and/or stop codons. Truncated copies included *atp6* (two truncated copies and one complete gene sequence), and *cox1*, *cob*, *nad2* and *atp9* (one truncated copy and one complete gene sequence). PCR amplifications were performed to confirm the presence of CMPCG copies in the *P. fijiensis* mitochondrial genome (Figure 2). The annotation showed that *cox 1* had one complete copy with an intron and a second truncated copy (see Figure 2A). Primers were designed to amplify three intra and intergenic regions in these two copies (Figure 2A–D). The amplified PCR bands were of the expected size showing that the assembly on this region was correct (Figure 2B). The presence of an intron in the complete cytochrome b (*cob*) gene of *P. fijiensis* was also experimentally confirmed by PCR amplification (Figure S1).

#### 3.1.2. Homing Endonucleases and Introns in the *P. fijiensis* Mitogenome

A total of two different mobile introns were annotated in the mitogenome of *P. fijiensis* (Figure 1A). All encoding introns were characterized as group I intron type, which encodes homing endonucleases (HE) [67]. They belonged to the LAGLIDADG family and were 680 bp for an intronic-ORF of 2968 bp length in *nad5* and 743 bp long for an intronic-ORF of 1056 bp length in *cob* (Figure 1, Table 2 and Table 3).

### 3.2. Comparative Analysis among Mitochondrial Genomes of Selected Mycosphaerellaceae

**Figure 3:**
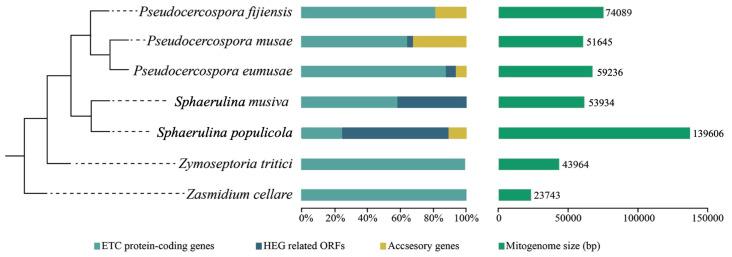
Comparison of mitochondrial genome sizes among Mycosphaerellaceae and contribution of, Conserved Mitochondrial Protein-Coding Genes (CMPCGs), hypothetical proteins, Homing Endonuclease Genes (HEGs) related Open Reading Frames (ORFs) and accessory genes, to genetic content of mitochondrial genomes. Mitochondrial genomes of Mycosphaerellaceae members are different in terms of genome size, and content of accessory genes and HEGs. Some genomes contain only CMPCGs while others exhibit HEG invasion. Phylogenetic relationships were inferred here.

The mitochondrial phylogeny of selected species of Mycosphaerellaceae confirms evolutionary relationships found in nuclear phylogenies (((*Sphaerulina musiva* + *Sphaerulina populicola*) + (*P. fijiensis* + (*P. eumusae* + *P. musae*)) *Zymoseptoria tritici*) *Zasmidium cellare*) [10,12] (Figure 3). We used this phylogeny to compare mitogenomes of *Pseudocercospora fijiensis*, *P. eumusae*, *P. musae*, *Sphaerulina populicola*, and *S. musiva*, and previously published mitogenomes of *Zymoseptoria tritici* and *Zasmidium cellare*. They showed a GC content of between 27% and 32% and a variability in genome size from 23,743 bp in “cellar mold” *Zasmidium cellare* to 136,606 bp in poplar pathogen *Sphaerulina populicola* (Table 2, Figure 3 and Figure S2).

**Figure 4:**
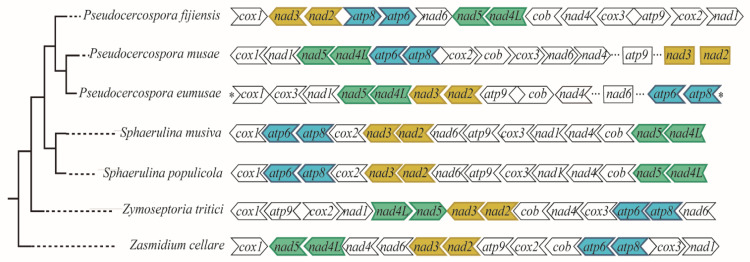
Comparison of Conserved Mitochondrial Protein Coding Genes (CMPCGs) order and orientation among selected Mycosphaerellaceae species. Gene order is not conserved across members even for closely related species except for *Sphaerulina* species. Colored gene pairs were always recovered as neighbors; asterisks indicate *atp8* is neighbor to *cox1* in *P. eumusae*. Ellipsis in *P. musae* and *P. eumusae* mean these genomes were both recovered in different contigs each (two and three respectively); where *nad6* and *atp9* could not be assembled to any contig we not complete a circular mitogenome.

Annotated genes of seven selected Mycosphaerellaceae mitogenomes showed the presence of a set of 14 Conserved Mitochondrial Protein-Coding Genes (CMPCGs), namely the subunits of the electron transport chain complex I (*nad1*, *nad2*, *nad3*, *nad4*, *nad4L*, *nad5* and *nad6*), complex III (*cob*), complex IV (*cox1*, *cox2* and *cox3*), and ATP synthase subunits (*atp6*, *atp8* and *atp9*) (Figure 4, Table S2). There was a small ribosomal subunit RNA (rns), a large ribosomal subunit rRNA (rnl), and a set of 24 to 38 tRNAs (Table 2).

In addition to these core genes, five out of seven mitogenomes also have hypothetical protein coding genes, accessory genes, additional copies of CMPCGs and genetic mobile elements (Figure 3, Table S2). Genome sizes, gene numbers and contents are heterogeneous among species (Table 2, Figure 3). All annotated genomes were found to have truncated gene duplications of some CMPCGs (Table S2). Alignments of truncated gene copies showed their sequences were not identical. However, one of the copies always had a complete coding sequence with a start and a stop codon (https://doi.org/10.6084/m9.figshare.13542191 (accessed on 10 February 2021)). Mitochondrial genomes from *P. musae* and *P. eumusae* also had RNA and DNA polymerases (Table S2).

**Figure 5:**
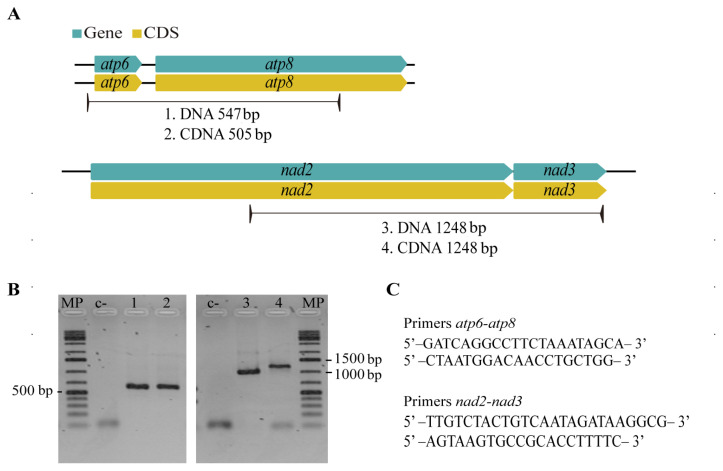
*P. fijiensis* RT-PCR assay to confirm bicistronic expression of genes. (**A**) Primers were designed between neighboring genes with hypothesized bicistronic transcription. (**B**) Gel results of RT-PCR assays for gene pairs *nad2-nad3*, *atp6-atp8* and its intergenic sequences, amplified bands have the expected sequence size. (**C**) Nucleotide sequences of designed primers.

The order of CMPCGs among selected Mycosphaerellaceae mitogenomes was variable, except for sister species *Sphaerulina populicola* and *S. musiva* (Figure 4). Despite this variability, gene pair order was always conserved for gene pairs *nad4L*-*nad5*, *nad3-nad2* and *atp8-atp6* among all mitogenomes (Figure 4). We mapped RNAseq assembled transcripts from transcriptomes available online for *S. musiva* (SRR1652271), *P. fijiensis* (SRR3593877, SRR3593879), *P. eumusae* (GDIK00000000.1) and *P. musae* (GDIN00000000.1) to *P. fijiensis*, *S. musiva*, *P. musae* and, *P. eumusae* mitochondrial genomes and found neighbor genes were always part of the same transcript for each species. This suggested that they were transcribed as a single mRNA. RT-PCR amplification and subsequent Sanger sequencing confirmed bicistronic expression for *nad3-nad2*, *atp8-atp6* gene pairs in *P. fijiensis* (Figure 5). 

Major differences in genome size among members of Mycosphaerellaceae seemed to be related to the invasive presence of HEG and ORFs in some mitogenomes. *Zasmidium cellare* and *Zymoseptoria tritici* have compact mitogenomes with core mitochondrial genes (CMPCGs) and lack of HEG. While, in other Mycosphaerellaceae, the presence of sequences containing LAGLIDADG or GIY-YIG domains related to Homing Endonuclease Genes (HEGs) was observed (Table 2). These ORFs ranged from one in *Pseudocercospora musae* and *P. eumusae* to 28 in *Sphaerulina populicola* (Table 2). *S. populicola* has the largest mitogenome report in this study (139,606 bp), suggesting HEGs might have caused fragmentation of CMPCGs. In almost all instances, pieces of CMPCGs were collinearly distributed and each fragment was found to be followed by an insertion of a HEG related sequence. An extreme case of HEG invasion to CMPCGs was found in *cox1* of *Sphaerulina populicola* (CDS: 1542 bp). This gene has twelve fragments distributed along 22,070 bp, each of them containing a piece of *cox1* followed by a HEG related domain (Figure S3). 

### 3.3. Inferred Mitochondrial Phylogeny and Diversification Times

**Figure 6:**
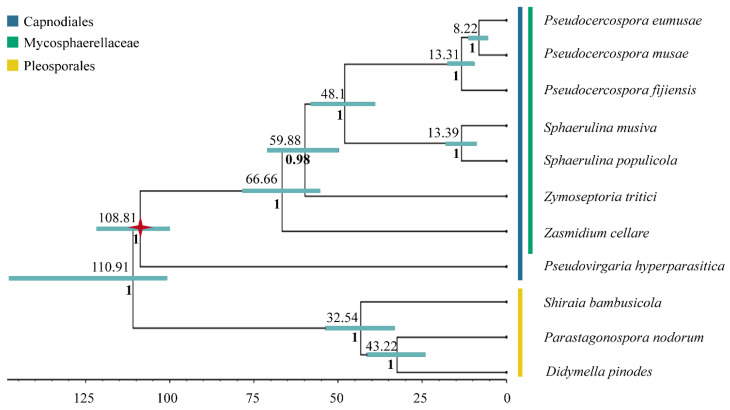
Mycosphaerellaceae Bayesian phylogeny and divergence time estimation. Mycosphaerellaceae Bayesian phylogeny with posterior probabilities in each branch and divergence time estimates calculated using fossil calibrations in BEAST2. Red star represents the fossil calibration placement.

A robust phylogeny with posterior probabilities greater than 0.97 was recovered, containing two main lineages (Pleosporales + Capnodiales). Divergence time estimates using fossil calibration are shown in Figure 6, with horizontal bars representing the 95% Highest Posterior Density (HPD) intervals for each node. According to our data, Mycosphaerellaceae diverged from the rest of Capnodiales at the end of the Mesozoic or the early Paleogene, about 66.66 MYA (55.47–78.27 MYA, 95% HPD). The earliest split within Mycosphaerellaceae gave rise to *Zasmidium cellare*. The species *Zymoseptoria tritici* diverged from (*Sphaerulina* + *Pseudocercospora*) also at the end of the Mesozoic or the early Paleogene 59.88 MYA (49.7–70.91 MYA, 95% HPD). The sister genera *Sphaerulina* + *Pseudocercospora* diverged in the Eocene, 48.1 MYA (39.34–57.86 MYA, 95% HPD). The sister clade to the species in the Sigatoka complex includes the species *Sphaerulina musiva* and *S. populicola* sharing their last common ancestor during the Miocene, 13.39 MYA (9.39–17.69 MYA, 95% HPD). The origin of *Pseudocercospora* members of the Sigatoka disease complex in bananas was dated to around 13.31 MYA (9.49–17.28 MYA, 95% HPD) during the Miocene while the sister *species P. eumusae* and *P. musae* split from their shared ancestor in the late Miocene 8.22 MYA (5.6–11.07 MYA, 95% HPD) (Figure 6).

Divergence times were also estimated using strict clock, in order to validate differences between mean node ages using relaxed lognormal clock versus strict clock. For a strict clock, 95% highest posterior density (HPD) intervals were significantly broader (27–131 MY) in comparison to (6–23 MY) and mean node ages were among 9–43 MY older (Table 3) (https://doi.org/10.6084/m9.figshare.13542938.v1 (accessed on 10 February 2021)).

## 4. Discussion

In this study, the complete mitochondrial genome of a plant pathogenic fungus, *Pseudocercospora fijiensis*, was sequenced and annotated. We also used comparative analysis and fossil calibration phylogenies to further understand the evolution of Mycosphaerellaceae mitogenomes. To date, more than 700 complete fungal mitogenomes are available online, but the mitogenomes of *Pseudocercospora* species have not been reported in the organelle genome database of NCBI (August 2017). The mitogenome of *P. fijiensis* and related species provides a molecular basis for further studies on molecular systematics and evolutionary dynamics of Ascomycota fungi especially belonging to Dothideomycetes. 

Ascomycetes mitochondrial genomes like most mitochondrial genomes along the tree of life generally consist of: two ribosomal subunits (*rnl* and *rns*), a distinct set of tRNAs and fourteen genes of the respiratory chain complexes (*cox1*, *cox2*, *cox3*, *cob*, *nad1* to *nad6*, *atp6*, *atp8* and *atp9*) [17]. The mtDNA of *P. fijiensis* contains the 14 mitochondrial inner membrane proteins involved in electron transport and coupled oxidative phosphorylation, as well as rnl and rns (Figure 1). These genes were also found in mitochondrial genomes of selected Mycosphaerellaceae species studied here. Additionally, DNA polymerases, RNA polymerases and Reverse transcriptases were found in *Pseudocercospora musae*, *P. eumusae* and *S. populicola* (Table S1). These polymerases and transcriptases might come from mitochondrial plasmids integrated into their mitochondrial genomes [21,68,69].

A variable number of open reading frames of unknown function and introns related to homing endonuclease genes (HEG), often including GIY-YIG or LAGLIDADG protein domains, were found in several Mycosphaerellaceae genomes including *P. fijiensis* (Table 2; Figure 1). Despite not being ubiquitous in Ascomycete mitogenomes these mobile elements are fairly common [16,17,22,67,70]. Differences in mitogenome sizes, gene order and gene duplication among Mycosphaerellaceae are attributed to heterogeneous content of accessory genes, and intron mobile sequences (Table 1, Figure 3 and Figure 4). 

The comparative study of Mycosphaerellaceae species selected mitogenomes showed that sizes and gene order are not conserved among members of the family (Figure 3 and Figure 4). Divergence times within sister clades *Pseudocercospora* 13.31 MYA (9.49–17.28 MYA, 95% HPD) and *Sphaerulina* 13.39 MYA (9.39–17.69 MYA, 95% HPD) are roughly the same (Table 3; Figure 6). But gene order in *Sphaerulina* species is conserved while in *Pseudocercospora* species it is not (Figure 4 and Figure 6). Nonetheless, some gene pairs were always found together, *atp6-atp8*, *nad2-nad3*, *nad4L-nad5*, in most studied species (Figure 4). Gene order variation among Mycosphaerellaceae could be due to mtDNA rearrangements caused by different processes, such as fusion, fission, recombination, plasmid integration and mobility [21]. Aguileta et al. (2014) compared 38 fungal mitogenomes to understand mitochondrial gene order evolution. They found evidence of gene rearrangements and a relationship with intronic ORFs and repeats. Their results support recombination in all fungal phyla. Despite rearrangements being pervasive in fungal mitogenomes, they found conserved gene pairs *nad2-nad3* and *nad4L-nad5* in most species [20]. Bicistronic transcription of *atp6-atp8*, *nad2-nad3* gene pairs was confirmed experimentally in *P. fijiensis* using RT-PCR (Figure 5). Maintenance of such proximity through evolution is important for mitochondria functions and modifications of such proximity can negatively affect organisms [71]. 

Even though sister species *Sphaerulina musiva* and *S. populicola* shared core gene content and gene order, their genomes sizes were quite different (53,234 bp and 139,606 bp). This could be due to a widespread occurrence of homing endonucleases HEG related to ORFs and accessory genes in the *S. populicola* mitogenome (Table S1, Figure S2). Mitogenome size variability due to the occurrence of mobile elements and accessory gene invasions was also observed in nine phylogenetically related species belonging to the genera *Aspergillus* and *Penicilium* [72]. In a phytopathogenic fungus *Sclerotinia borealis* mitogenome size expansion was also shown to be due to plasmid-like sequences and HEGs related to ORFs [73].

Mitochondrial gene duplication is seldom described in fungi. HEGs invasion has been previously described in fungal mtDNA, where truncated genes were ubiquitous [74]. *P. fijiensis* and other Mycosphaerellaceae mitogenomes have 2 to12 truncated copies of Conserved Mitocondrial Protein Coding Genes (CMPCGs) (Table S1). Truncated CMPCGs have partial sequences lacking start and/or stop codons. Truncated gene copies did not have a particular distribution pattern; they were either close to each other or dispersed in the genomes. Mardanov et al. (2014) found duplications of truncated extra copies of *atp9* and *atp6* in the phytopathogenic fungus *Sclerotinia borealis* [73]. Two incomplete copies of *atp6* were also found on different strands of the mtDNA of *Shiraia bambusicola* (Pleosporales) [46]. However, it is not common to find highly fragmented genes such as the *cox1* of *Sphaerulina populicola* (Figure S3). A similar case was reported in the mitogenome of *Sclerotinia borealis*, where thirteen introns of *cox1* and truncated copies of CMPCGs were found [73].

Fossil calibrated phylogenies for the Mycospharellaceae had later diversification times 66.66 MYA (55.47–78.27 MYA, 95% HPD) compared to previous studies 186.7–143.6 MYA [10]. The Sigatoka disease complex had an early divergent *Pseudocercospora fijiensis*, that splits from sister species *P. musae* + *P. eumusae* 13.31 MYA (9.49–17.28 MYA, 95% HPD); while sister species *P. eumusae* and *P. musae* split from their shared ancestor in the late Miocene 8.22 MYA (5.6–11.07 MYA, 95% HPD) (Figure 6). Chang et al. (2016) estimated the divergence of *P. fijiensis* from their last common ancestor with *P. musae* + *P. eumusae* to be between 39.9–30.6 MYA and the divergence of *P. musae* and *P. eumusae* to be between 22.6–17.4 MYA. Diversification ages estimated here were based on mitochondrial markers and Bayesian analysis using both relaxed and strict clock models. These have placed all diversification times in the Mycosphaerellaceae at later times than those calculated by Chang et al. (2016) using nuclear markers, penalized maximum likelihood analysis and strict clock, except for that of *Sphaerulina* (Table 3).

These differences in diversification times compared to Chang et al. (2016) could be due to different calibration points and dating methods. Chang et al. (2016) implemented r8s Likelihood methods [75] and a calibration at the origin of the Dothideomycetes crown group (394–285 MYA) using previous Bayesian estimations [76]. For this study Bayesian analysis in BEAST2 [60] using fossil calibration with a Metacapnodiaceae fossil was implemented [64]. We found that Sigatoka disease members (13.31 MYA (9.49–17.28 MYA, 95% HPD)) appeared after the genus *Musa* (27.9 MYA (21.5–34.4, 95% HPD)) [77]. Species member of this genus within the section Eumusa *sensu latto* comprise cultivated banana and are the host of the Sigatoka disease complex. This naturally prompts a further question: did the Sigatoka disease complex originate through host-tracking evolution? This hypothesis explains that a pathogen is likely to be younger than the host until changes related to the genetics of the pathogens or/and exogenous factors have observed alterations in their virulence spectra [70]. We are currently limited in terms of a good taxonomic sampling and biogeographical analysis for *Pseudocercospora* species in arriving at an answer to this question. A host tracking coevolution hypothesis has also been proposed for *Zymoseptoria tritici* (syn. *Mycosphaerella graminicola*) [5].

## 5. Conclusions

We successfully sequenced and analyzed mitochondrial genomes of *Pseudocercospora fijiensis, P. eumusae, P. musae, Sphaerulina populicola* and *S. musiva.* A robust mitochondrial phylogeny containing two main lineages (Pleosporales + Capnodiales) was obtained and divergence times were estimated using fossil calibration. Fossil calibrated phylogenies are reported for the first time here for fungal plant pathogens that had later diversification times for the origin of all the species involved, compared to previous studies. Genome size variation and organization among Mycosphaerellaceae could be related to the proliferation of type I and II introns, gene duplications and possible plasmid insertions, phenomena known for many fungal mitogenomes. Despite their order variability, some genes were always recovered as neighbors in all mitogenomes analyzed. Bicistronic expression for *nad3-nad2*, *atp8-atp6* gene pairs in *P. fijiensis* was confirmed experimentally. Further gene editing and virulence assays will be important to shed light on fungal adaptation and more effective disease control strategies. Phylogenomic studies including a good taxonomic sampling and biogeographical analysis for *Pseudocercospora* species will further clarify whether the Sigatoka disease-causing species virulence flared-up after *Musa* domestication.

## Figures and Tables

**Table 1:** Characteristics and organization of annotated genes in the mitogenome of *P. fijiensis.*

Gene	Start Position	Stop Position	Length (bp)	Direction	Start Codon	Stop Codon	GC Contents	Product
nad6	435	1109	675	forward	ATA	TAA	23%	subunit 6 of NADH dehydrogenase
nad5	1922	7012	5091	reverse	ATA	TAA	24.8	NADH dehydrogenase subunit 5
ORF1	3253	5001	1749	reverse	ATG	TAA	22.8	hypothetical protein
LAGLIDADG	5042	5722	681	reverse	TTA	TAA	24.4	LAGLIDADG domain containing endonuclease
nad4L	7009	7278	270	reverse	TTA	TAA	23.7	NADH dehydrogenase subunit 4L
trnN(gtt)	9370	9440	71	reverse	-	-	-	tRNA-Asn
trnP(tgg)	9480	9552	73	reverse	-	-	-	
ORF2	9600	10271	672	reverse	ATG	TAA	29.9	hypothetical protein
ORF3	11452	11973	522	forward	TTA	TAG	26.2	hypothetical protein
ORF4	12481	12969	489	forward	ATG	TAA	33.5	hypothetical protein
ORF5	14399	15151	753	reverse	ATA	TAA	-	hypothetical protein
Large subunit rRNA	15433	21413	5981	forward	-	-	-	large subunit ribosomal RNA
ORF6	16699	17037	339	forward	ATT	TAA	14.7	hypothetical protein
cob	23991	26209	2219	reverse	TTA	TAA	29.3	cytochrome b
LAGLIDADG	24749	25492	744	reverse	-	-	-	LAGLIDADG domain containing endonuclease
trnH(gtg)	26876	26948	73	reverse	-	-	-	tRNA-His
trnQ(ttg)	26974	27046	73	reverse	-	-	-	tRNA-Gln
trnL1(tag)	29371	29454	84	reverse	-	-	-	tRNA-Leu
trnF(gaa)	29531	29603	73	reverse	-	-	-	tRNA-Phe
trnA(tgc)	30770	30841	72	reverse	-	-	-	tRNA-Ala
trnE(ttc)	31173	31244	72	reverse	-	-	-	tRNA-Glu
trnL2(taa)	31253	31335	83	reverse	-	-	-	tRNA-Leu
trnM(cat)	31478	31550	73	reverse	-	-	-	tRNA-Met
trnM(cat)	31555	31625	71	reverse	-	-	-	tRNA-Met
trnT(tgt)	31714	31784	71	reverse	-	-	-	tRNA-Thr
trnR(tct)	32001	32071	71	reverse	-	-	-	tRNA-Arg
trnC(gca)	32105	32175	71	reverse	-	-	-	tRNA-Cys
trnR(acg)	32212	32290	79	reverse	-	-	-	tRNA-Arg
nad4	32730	34220	1491	reverse	ATA	TAA	25.4	NADH dehydrogenase subunit 4
ORF7	34319	35902	1584	reverse	ATG	TAA	22.9	hypothetical protein
trnR(cct)	36840	36910	71	reverse	-	-	-	tRNA-Arg
trnS2(tga)	37775	37862	88	reverse	-	-	-	tRNA-Ser
trnI(gat)	37911	37982	72	reverse	-	-	-	tRNA-Ile
trnW(tca)	38019	38090	72	reverse	-	-	-	tRNA-Trp
trnS1(gct)	38154	38234	81	reverse	-	-	-	tRNA-Ser
trnD(gtc)	38279	38351	73	reverse	-	-	-	tRNA-Asp
trnG(tcc)	38481	38551	71	reverse	-	-	-	tRNA-Gly
ORF8	38798	39094	297	forward	ATG	TAG	19.5	hypothetical protein
cox3	43007	43816	810	reverse	ATG	TAA	31.7	Cytochrome c oxidase subunit III
atp9	44999	45223	225	reverse	ATG	ATG	40.9	ATP Synthase Membrane Subunit 9
trnK(ttt)	45506	45578	73	reverse	-	-	-	tRNA-Lys
trnV(tac)	45607	45679	73	reverse	-	-	-	tRNA-Val
trnM(cat)	45915	45987	73	reverse	-	-	-	tRNA-Met
cox2	46989	47738	750	reverse	TAA	TAA	29.9	cytochrome c oxidase subunit II
nad1	50440	51600	1161	forward	ATG	TAA	30	NADH dehydrogenase subunit 1
Small subunit rRNA	51849	53773	1925	forward	-	-	-	small subunit ribosomal RNA
trnY(gta)	53797	53881	85	forward	-	-	-	tRNA-Tyr
cox1	55837	57708	1872	forward	TTA	TAG	32.2	cytochrome c oxidase subunit I
ORF cytb-like	59442	59672	231	reverse	TTA	TAA	22.5	cytb-like ORF
trnH(gtg)	60291	60363	73	reverse	-	-	-	tRNA-His
trnQ(ttg)	60389	60461	73	reverse	-	-	-	tRNA-Gln
ORF9	60802	61359	558	forward	ATG	TAA	35.1	hypothetical protein
trnF(---)	64038	64111	74	reverse	-	-	-	tRNA-Phe
trnA(tgc)	64155	64226	72	reverse	-	-	-	tRNA-Ala
trnE(ttc)	64498	64569	72	reverse	-	-	-	tRNA-Glu
ORF10	64714	65247	534	reverse	ATA	TAG	27	hypothetical protein
trnI(tat)	65672	65744	73	reverse	-	-	-	tRNA-Ile
trnM(cat)	65749	65819	71	reverse	-	-	-	tRNA-Met
trnT(tgt)	65908	65978	71	reverse	-	-	-	tRNA-Thr
trnR(tct)	66182	66252	71	reverse	-	-	-	tRNA-Arg
trnC(gca)	66286	66356	71	reverse	-	-	-	tRNA-Cys
trnR(acg)	66393	66465	73	reverse	-	-	-	tRNA-Arg
ORF11	68278	69276	999	reverse	ATG	TAA	21.5	hypothetical protein
trnG(tcc)	69523	69593	71	forward	-	-	-	tRNA-Gly
nad3	69768	70139	372	reverse	ATG	TAA	27.2	NADH dehydrogenase subunit 3
nad2	70140	71819	1680	reverse	ATG	TAA	24.5	NADH dehydrogenase subunit 2
atp8	72609	72758	150	forward	ATT	TAA	24	ATP Synthase Subunit 8
atp6	72801	73589	789	forward	ATA	TAA	27.8	ATP Synthase Subunit 6

**Table 2:** Comparison of mitogenomes of *Pseudocercospora fijiensis* and closely related species (*Sphaerulina musiva*, *S. populicola*, *P. musae*, *P. eumusae* and *Zasmidium cellare*, *Zymoseptoria tritici*). Intragenic (genic) regions include regions of standard CMPCGs (Conserved Mitochondrial Protein Coding Genes), open reading frame (ORFs), rRNAs, and tRNAs. Intergenic regions include regions among standard CMPCGs, ORFs, rRNAs, and tRNAs.

Item	*P. musae*	*P. eumusae*	*P. fijiensis*	*S. musiva*	*S. populicola*	*Z. cellare*	*Z. tritici*
						
Genome size (bp)	51,645	59,236	74,089	53,234	139,606	23,743	43,964
GC content (%)	27.80	27	19.2	24.5	31.70	27.80	31.90
No. of introns	1	1	2	6	28	0	0
No. of standard Protein Coding Genes (CMPCGs)	14	14	14	14	14	14	14
No. of rRNAs	2	2	2	2	2	2	2
No. of tRNAs	24	25	38	29	29	25	27
Genic regions (%)	63.25	40.73	65.55	65.06	98.25	79.39	67.79
Intergenic regions (%)	36.75	59.27	46.45	34.94	1.75	20.51	32.21
Number of GIY-YIG intragenic regions	0	0	0	1	5	0	0
Number of GIY-YIG intergenic regions	1	1	0	0	2	0	0
Number of LAGLIDADG intragenic regions	0	0	0	3	18	0	0
Number of LAGLIDADG intergenic regions	0	0	2	1	3	0	0
Number of Repetitive Sequences	32	43	29	8	20	2	0

**Table 3:** Comparison between divergence time of some clades among different studies.

	Present Study		Chang et al. 2016
*Molecular Clock*	*Relaxed-Log normal*	*Strict clock*	*Strict clock*
Type of Data	13 mitochondrial protein-coding genes	13 mitochondrial protein-coding genes	46 nuclear single-copy genes
Divergence Time estimation method	Bayesian analysis in BEAST2 v2.5.1	Bayesian analysis in BEAST2 v2.5.1	Penalized likelihood analysis in the program r8s v1.7
Fossil calibration	Capnodiales-Metacapnodiaceae Fossil	Capnodiales- Metacapnodiaceae Fossil	Dothideomycetes crown group
Capnodiales	108.81 (100.17–121.35 MYA, 95% HPD)	151.96 (101.04–232.179 MYA, 95% HPD)	234.2–180.2 MYA
Mycosphaerellaceae	66.66 (55.47–78.27 MYA, 95% HPD)	97.24 (64.27–155 MYA, 95% HPD)	186.7–143.6 MYA
*Sphaerulina*	13.39 (9.39–17.69 MYA, 95% HPD)	30.31 (19.69–48.63 MYA, 95% HPD)	near 10 MYA
*Pseudocercospora + Sphaerulina*	48.1 (39.34–57.86 MYA, 95% HPD)	84.46 (55.6–134.54 MYA, 95% HPD)	146.6–112.8 MYA
*Pseudocercospora*	13.31 MYA (9.49–17.28 MYA, 95% HPD)	27.66 MYA (17.8–44.27MYA, 95% HPD)	39.9–30.6 MYA
*P. eumusae + P. musae*	8.22 MYA (5.6–11.07 MYA, 95% HPD)	17.87 MYA (11.5–28.71 MYA, 95% HPD)	22.6–17.4 MYA

## Data Availability

*Pseudocercospora fijiensis* NADH dehydrogenase subunit 5 (*nad5*) mRNA, partial cds; mitochondrial 502 bp linear mRNA MN171340.1 GI:1817958850. *Pseudocercospora fijiensis* cytochrome b (*cob*) mRNA, partial cds; mitochondrial, 159 bp linear mRNA MN171339.1 GI:1817958848. *Pseudocercospora fijiensis* cytochrome b (*cob*) gene, partial cds; mitochondrial, 1121 bp linear DNA MN171338.1 GI:1817958846. *Pseudocercospora fijiensis* NADH dehydrogenase subunit 2 (*nad2*) and NADH dehydrogenase subunit 3 (*nad3*) mRNAs, partial cds; mitochondrial, 2377 bp linear mRNA MN171337.1 GI:1817958843. *Pseudocercospora fijiensis* NADH dehydrogenase subunit 3 (*nad3*) and NADH dehydrogenase subunit 2 (*nad2*) genes, partial cds; mitochondrial, 1188 bp linear DNA, MN171336.1 GI:1817958840. *Pseudocercospora fijiensis* ATP synthase subunit 8 (*atp8*) gene, complete cds; and ATP synthase subunit 6 (*atp6*) gene, partial cds; mitochondrial, 701 bp linear DNA, MN171335.1 GI:1817958837. *Pseudocercospora fijiensis* ATP synthase subunit 8 (*atp8*) and ATP synthase subunit 6 (*atp6*) mRNAs, partial cds; mitochondrial, 676 bp linear mRNA, MN171334.1 GI:1817958834. Arcila Galvis, Juliana Estefanía; Arias, Tatiana (2020): Data non-curated for NCBI *Pseudocercospora fijiensis* and related Mycosphaerellaceae mitochondrial genomes. Figshare Dataset: https://figshare.com/account/home#/projects/78525 (accessed on 10 February 2021). Arcila Galvis, Juliana Estefanía; Arias, Tatiana (2020): Annotated mitochondrial genomes of Mycosphaerellaceae. Figshare Dataset: https://doi.org/10.6084/m9.figshare.12101058.v1 (accessed on 10 February 2021). Arcila Galvis, Juliana Estefanía; Arias, Tatiana (2020): *P.fijiensis* DATA. Figshare Dataset: https://doi.org/10.6084/m9.figshare.12101013 (accessed on 10 February 2021). Arcila Galvis, Juliana Estefanía; Arias, Tatiana (2020): xml files used to build Bayesian phylogenies. figshare. Figshare Dataset. https://doi.org/10.6084/m9.figshare.12101055.v1 (accessed on 10 February 2021).

## Supplementary Materials

The following are available online at https://www.mdpi.com/2075-1729/11/3/215/s1: Table S1. Sources of genomic data used in this study. Table S2: Protein-coding genes annotated in Mycosphaerellaceae mitochondrial genomes, Figure S1: Mitochondrial gene duplication in *Pseudocercospora fijiensis*. A. The intron of *cob*. B. PCR amplification of intra and intergenic sequences among exons. C. Primers used for the amplification of *cob*, Figure S2: Circular map of the mitogenome of *Sphaerulina musiva* and *Sphaerulina populicola*. Smaller 53,234 bp and larger 139,606 bp mitogenomes from selected species of Mycosphaerellaceae used in this study, Figure S3: Homing endonuclease invasion in the mitochondrial genome of *Sphaerulina populicola*. Truncated *cox1* copy possibly due to a pervasive HEGs invasion. Numbers one-12 represent different internal Open Reading Frames (ORFs). Domains content in the ORFs are listed in the table along with their position within *cox1*.
